# Adsorption of basic yellow dye dataset using Ethiopian kaolin as an adsorbent

**DOI:** 10.1016/j.dib.2019.104504

**Published:** 2019-09-13

**Authors:** Tadele Assefa Aragaw, Fikiru Temesgen Angerasa

**Affiliations:** Faculty of Chemical and Food Engineering, Bahir Dar Institute of Technology, Bahir Dar University, Ethiopia

**Keywords:** Kaolin adsorbents, Adsorption process, Operation conditions, Basic yellow dye

## Abstract

This article presents batch experimental data describing the main batch adsorption operation parameters. Also the adsorption models (adsorption isotherm, adsorption kinetics and thermodynamic studies) of basic yellow dye on to the raw and treated kaolin adsorbents. Besides, instrumental analyses were recorded to characterize kaolin adsorbent. Such as, thermogravimetric analyzer, Fourier transformation infrared and scanning electroscope with energy dispersion spectroscopy were used. UV–Visible spectrometer was used throughout the experimental study for the determination of absorbance. The effect of adsorption temperature (30 °C, 50 °C 70 °C), PH (3, 7, 9), initial dye concentration (20 mg/l, 40 mg/l, 60 mg/l), contact time (20 min, 40 min, 60 min, 80 min, 100 min) and adsorbent dosage (0.1, 0.5, 1, 1.5, 2 g/100ml) have been well determined. For adsorbent characteristics, we provide dataset regarding (i) thermogravimetery with differential scanning calorimetery, (ii) Fourier transform infrared spectral data before and after basic yellow dye adsorption process, (iii) scanning electroscope with energy dispersion spectroscopy image at ×500 resolution, (iv) X-ray diffraction and, (v) batch adsorption experimental parameters records. Regarding scanning electroscope with energy dispersion spectroscopy image, we provide data of three surface morphology image and three elemental distribution spectra for raw and treated kaolin adsorbent.

**Table:** Specifications Table

Subject	Environmental Science
Specific subject area	Adsorptions of dye wastewater using kaolin adsorbent
Type of data	Table Image Figure
How data were acquired	UV–Visible spectrophotometer, FTIR, SEM, TGA-DSC, X-ray diffraction (XRD) Origin software
Data format	Raw and Processed
*Experimental Factors*	3 Adsorption temperature (30, 50 and 70°C) 3 Initial dye solution concentration (20, 40, and 60 mg/l) 3 Solution pH (3, 7, and 9) Using the optimum values of the above, 5 contact time (20, 40, 60, 80, and 100 min) and 5 adsorbent dosage (0.1, 0.5, 1, 1.5 and 2g/100ml) were investigated.
*Data Source Location*	Institution: Bahir Dar Institute of Technology City/Town/Region:Debre Tabor and Bahir Dar Country: Ethiopia Latitude and longitude; and GPS coordinates 11.5742° N, 37.3614° E and 11°35′37.10″ N 37°23′26.77″ E respectively for Bahir Dar. Latitude and longitude; and GPS coordinates 11.8567° N, 38.0155° E and 11.8499966 38.0166666 respectively for Debre Tabor
*Data Accessibility*	Repository name: [**Mendeley Data**] Data identification number: [10.17632/cncmbh6gb8.5] Direct URL to data: [https://data.mendeley.com/datasets/cncmbh6gb8/5]

**Table:** 

**Value of the Data** • The physicochemical characteristics are input to know the performance of kaolin adsorbents, data of operating parameters are used to decide optimum value and the models are used to describe the behavior of batch experimental data. • The dataset can be used for industries to implement kaolin adsorbent as cost effective and environmental friendly for dye wastewater treatment and researchers in the university can be used a pre-experiment for other researches. • The dataset can be used for removal of other types of dyes (reactive dyes, acidic dyes etc.) from wastewater. • Generally, these data can be used to design adsorption process to remove dye ions from dye containing wastewater.

## 1 Data

We report on several batch experimental study and adsorption models on the potential adsorption capacity of kaolin adsorbents datasets of three adsorbent at different operation parameters and model. Six kinds of data for basic yellow dye removal are reported: batch experiment datasets (sheet 1 from excel file), Optimization datasets (sheet 2 from excel file), Isotherm, kinetics and Thermodynamics dataset (sheet 3 from excel file), thermogravimetric with Differential Scanning Calorimetry (sheet 4 from excel file), Fourier transform infrared, before and after adsorption for basic yellow dye, dataset (sheet 5 from excel file), X-ray Diffraction (sheet 6 from excel file) and Scanning electron microscopy with energy dispersive spectroscopy dataset for beneficiated, calcined and raw kaolin adsorbents (images from three separate word file).

## 2 Experimental design, materials, and methods

### 2.1 Material and chemicals

Natural kaolin was collected from local area, Debre Tabor Town, Amhara Region, Ethiopia. Synthetic solution was prepared from Basic yellow dye. All laboratory grade reagents, sodium hydroxide (NaOH), Hydrochloric acid (HCl), potassium bromide (KBr), distilled water and liquid nitrogen was used without any purification.

### 2.2 Preparation of adsorbent

Collected kaolin was crushed, milled and screened using jaw crusher, disc mill and Standard sieves, respectively. The size of kaolin powder was sieved to 75 μm. The powder of kaolin was calcined at 700°C using Muffle Furnace. Additionally, the powder of kaolin was beneficiated using distilled water in the conical flask of 1L capacity. Finally, three types of kaolin adsorbents were prepared from natural kaolin i.e. raw, beneficiated and calcined kaolin adsorbent in order to investigate their efficiency for dye adsorption from aqueous solution.

### 2.3 Preparation of stock dye solution

Basic yellow dye was taken from Bahir Dar Textile Share Company, Amhara Region, Ethiopia. The physical state of basic yellow dye is powdered solid. Basic yellow dye has the chemical formula; C_21_H_27_N_3_O_5_S as well as its molecular weight and maximum wave length are 433.52 g/mol and 438 nm, respectively. The stock solution (500 mg/L) of the dye was prepared by dissolving 0.5 g of basic yellow dye in 1 L of distilled water and required concentrations were obtained by dilution of the stock solution. The maximum wave length (438 nm) is obtained after scanning of the sample of dye using UV/VIS spectrometer (Lamda 35 Ferkin Elmer).

### 2.4 Experimental design and description

A measure amount of distilled water (100ml) was taken in 250 ml of conical flask for batch experiment. In conical flask calculated amount of initial dye concentration and adsorbent were added and agitated with magnetic stirrer on digital hot plate at 200 rpm. The initial pH of solution was adjusted with 1M HCl or 1M NaOH before adding the adsorbent. The batch adsorption experiments were performed for a wide range of initial dye concentration (20, 40 and 60 mg/L), temperature (30, 50 and 70°C), solution pH (3, 7 and 9), adsorbent dosage (0.1, 0.5, 1, 1.5, and 2 g) and contact time (20, 40, 60, 80 and 100 min). At the end of each experiment, small amount of the solutions was withdrawn at predetermined time and filtered. The absorbance value of a solution was measured after adsorption experiment for each run. The finial dye concentration was calculated from calibration curve. The removal efficiency of the dye was calculated by Equation (1) [1]. The equilibrium state concentration (loading) of adsorbate in the solid phase (qe, mg/g) and concentration (loading) of adsorbate in the solid phase at any time (qt,mg/g) were determined by Equations (2) and (3), respectively, [2].

(1) $$\text{Removal}\;\text{Efficiency}\;\text{(\%)}=\frac{\left({\text{C}}_{\text{o}}-{\text{C}}_{\text{t}}\right)}{{\text{C}}_{\text{o}}}\times 100$$

(2) $${\text{q}}_{\text{e}}=\frac{\text{V}\left({\text{C}}_{\text{o}}-{\text{C}}_{\text{e}}\right)}{\text{m}}$$

(3) $${\text{q}}_{\text{t}}=\frac{\text{V}\left({\text{C}}_{\text{o}}-{\text{C}}_{\text{t}}\right)}{\text{m}}$$

Where, C_o_ is the initial dye concentration (mg/L), C_e_ is a solute (dye) concentration in the liquid phase at the equilibrium (mg/L), C_t_ is the dye concentration in liquid phase at any time (mg/L), m is the amount of adsorbent (g) and V is the volume of solution (L).

### 2.5 Analysis and characterization of kaolin adsorbents

**Fig. 1:**
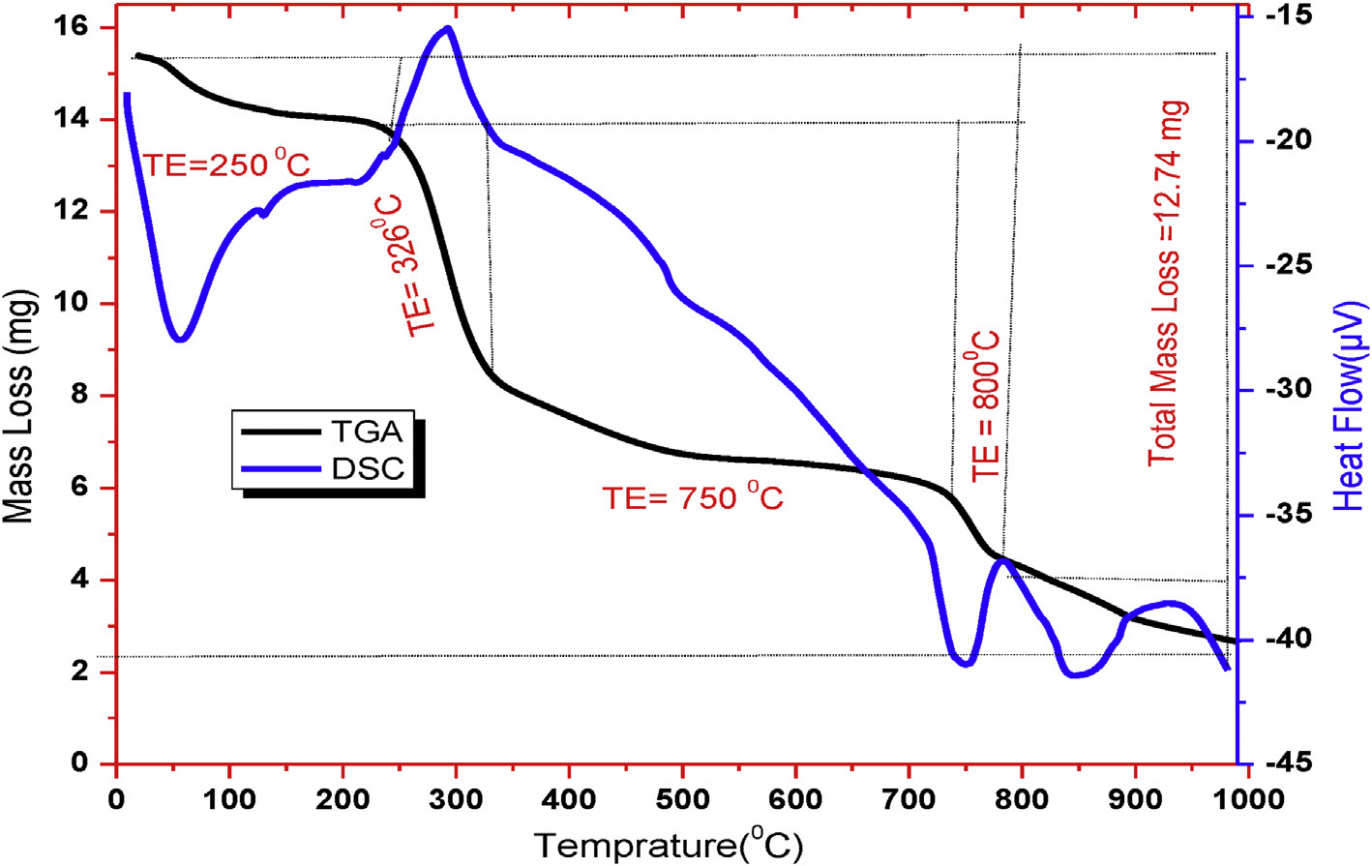
Thermogravimetric analysis of kaolin adsorbent.

**Fig. 2:**
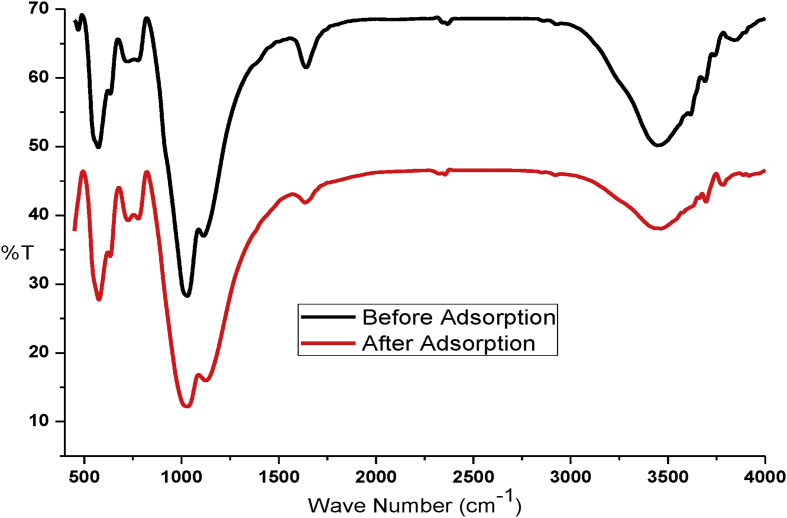
FTIR analysis of kaolin adsorbents before and after adsorption.

**Fig. 3:**
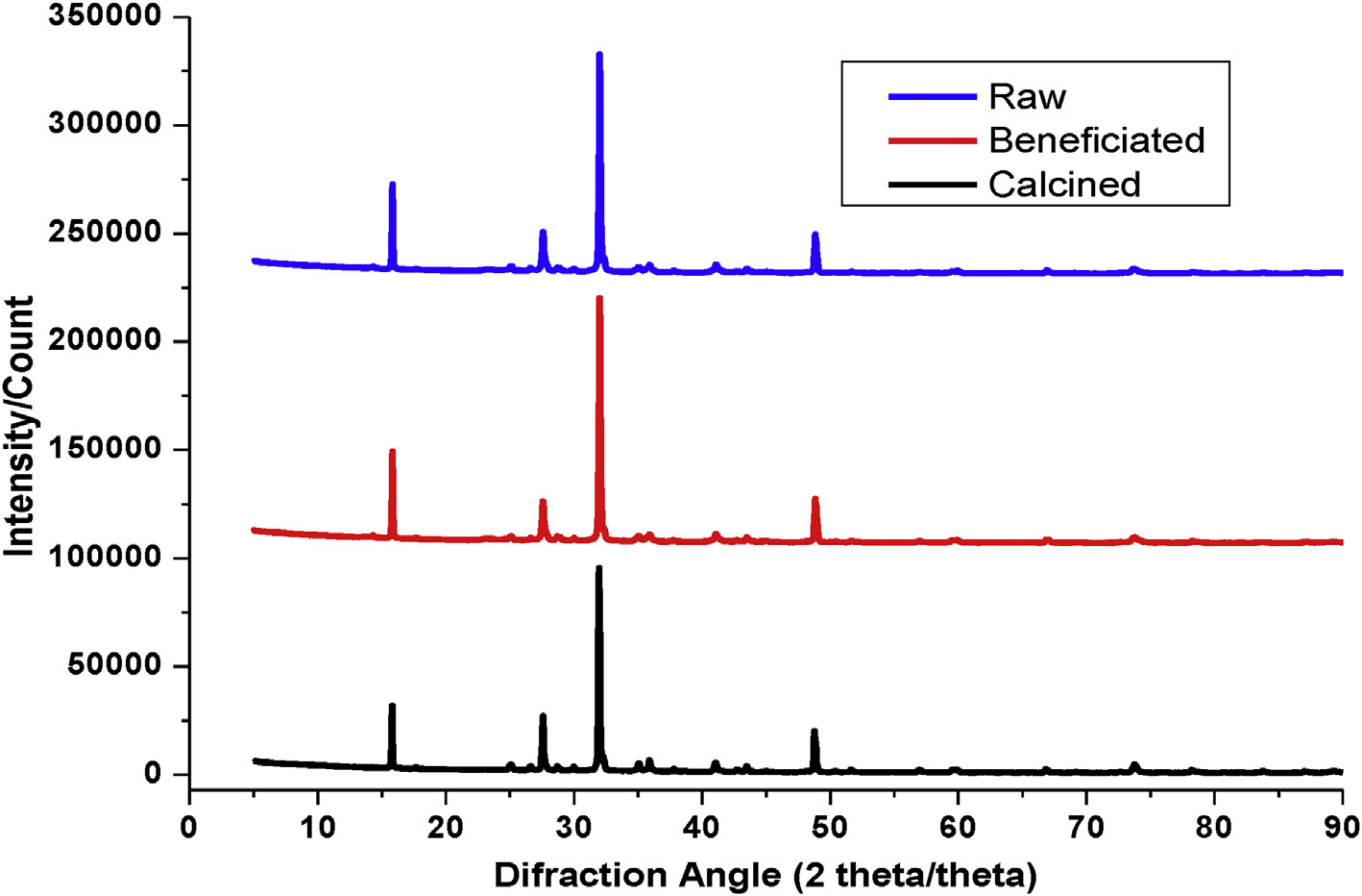
XRD pattern for raw, beneficiated and calcined kaolin adsorbents.

**Fig. 4:**
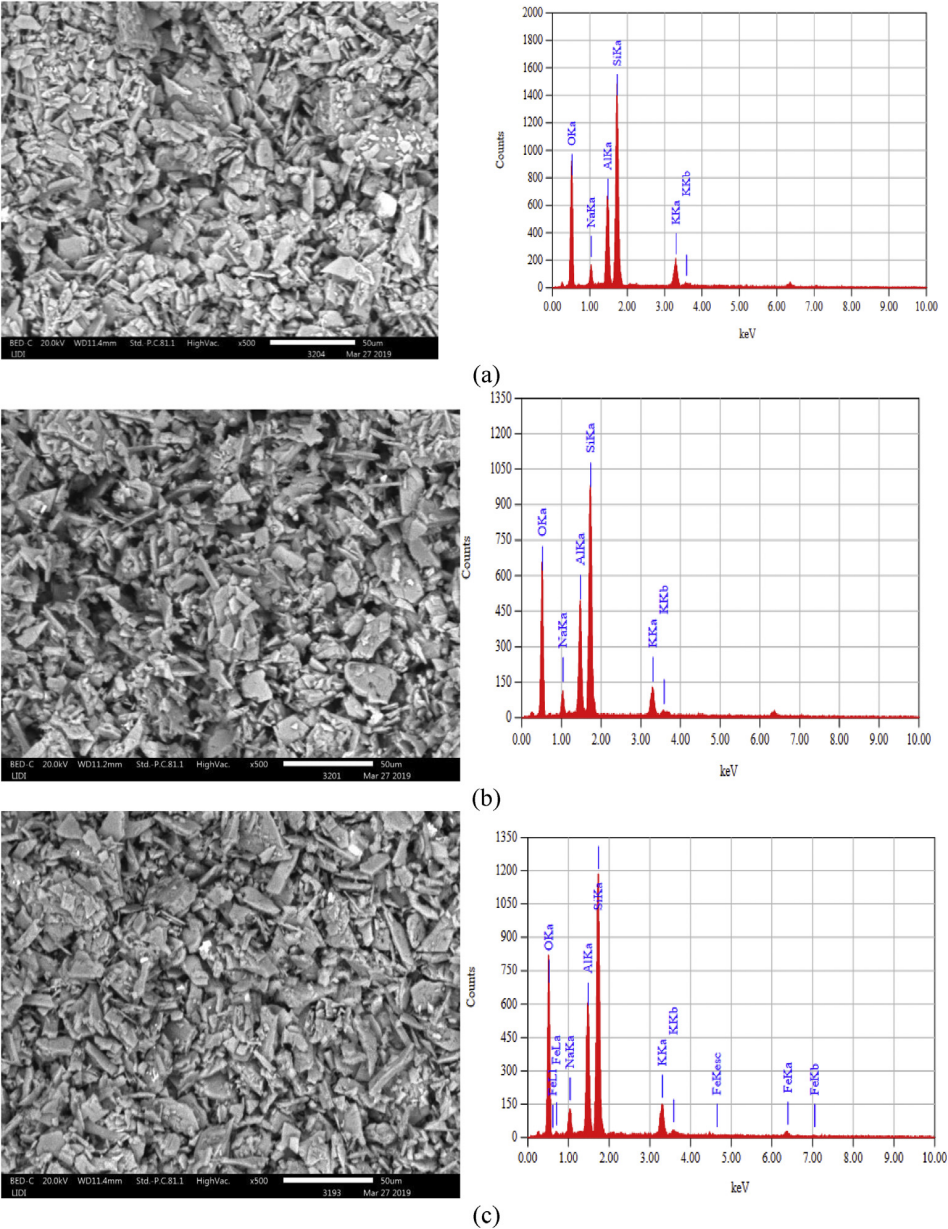
SEM/EDS image of a) raw b) beneficiated and c) calcined kaolin.

The dye absorbance values were measured by UV/VIS spectrometer (Lamda 35 Ferkin Elmer) at a maximum wave length (438 nm). The thermogravimetric properties is important because metakaolin is not a simple mixture of amorphous silica and alumina, but rather a complex amorphous structure that maintains some longer-range order due to the stacking of its hexagonal layers [3]. Mass losses and thermal property of kaolin adsorbent were analyzed by Thermogravimeter (SDT Q600) with liquid nitrogen from 20 to 1000°C temperature. The processed data of TGA-DSC is presented in Fig. 1. The powder of kaolin was calcined at 700°C using Muffle Furnace (Nabertherm B180). Fourier transform infrared (FTIR) spectra were obtained in the range of 400–4000 cm^−1^ to analyze the surface function group and their percent transmittance by using a PerkinElmer Frontier Spectrometer (ILC38B6PD7) before and after adsorption. The processed data is presented in Fig. 2. The Qualitative and quantitative characteristic of the phases, its crystallinity and the number of phases that is present in were determined by X-ray diffractometer (MIN 3740) with a continuous scanning scan axis of 2θ/θ about a full scan of 6474 and scanning range from 4.99 to 90. The raw data is presented in mendeley data from excel file and the processed data is presented in Fig. 3. Surface morphology of kaolin adsorbents was analyzed by using Scanning Electron Microscopy (IT300 LA). The image of SEM with the corresponding EDS is presented in Mendeley Data of word file and also presented in Fig. 4 for the three samples.

### 2.6 Adsorption isotherm model

The raw data of isotherm was presented in Mendeley Data of Microsoft Excel in worksheet 3. Langmuir and Freundlich isotherm models were used to determine the relationship between dye ions adsorbed on the kaolin adsorbents surfaces and dye ions remaining in the solution. The Langmuir isotherm model assumes that sorption occurs in certain places and within the sorbent. In addition, the model assumes that sorption of dye ions occurs within homogeneous monolayers without any interactions between dye ions at the sorbent surface. The Freundlich isotherm model is an experimental model and assumes that the sorption of dye ions occurs at heterogeneous surfaces and active sites with different energies. The Langmuir and Freundlich model linear equation were presented in Eqs. (4), (5), respectively [4], [5].

(4) $$\frac{{\text{C}}_{\text{e}}}{{\text{q}}_{\text{e}}}=\frac{{\text{C}}_{\text{e}}}{{\text{q}}_{\text{m}}}+\frac{1}{{\text{q}}_{\text{m}}{\text{K}}_{\text{L}}}$$

(5) $$\text{Log}\;{\text{q}}_{\text{e}}=\log\;{\text{k}}_{\text{f}}+\frac{1}{\text{n}}\log\;{\text{C}}_{e}$$

Where q_m_ is sorption capacity (mg/g), K_L_ is sorption energy (L/g), Kf and n are the Freundlich model constants and indicating the relationship between sorption capacity and sorption intensity, respectively. If n = 1, n > 1, and n < 1, then the sorption process would be the linear, physical, or chemical in its nature, respectively.

The calculated Langmuir and Freundlich constant parameters from raw data of Mendeley Data of Microsoft Excel in worksheet 3 were presented in Table 1.

**Table 1:** Langmuir and Freundlich isotherm Parameters for adsorption for basic yellow dye.

Types of kaolin adsorbents	Langmuir Isotherm Parameters			Freundlich Isotherm Parameters		
	q_m_ (mg/g)	K_L_ (L/mg)	R^2^	K_f_ (mg/g)	n	R^2^
Beneficiated	2.174	2.190	0.988	1.309	3.571	0.999
Raw	1.818	2.594	0.999	1.236	2.994	0.974
Calcinated	0.885	4.105	0.998	1.115	2.849	0.971

### 2.7 Adsorption kinetic model

The raw data of kinetic was present in Mendeley Data of Microsoft Excel in worksheet 3. Kinetic model was used to know the information about experimental data of adsorption mechanism as a function of mixing time. The raw data of effect of mixing time on adsorption process was presented Mendeley Data of Microsoft Excel in worksheet 2. Pseudo first-order and pseudo second-order were used to analyze kinetic model. The linear equations for both models were presented in Eqs. (6), (7), respectively [6].

(6) $$\text{log}{\text{(q}}_{\text{e}}-{\text{q}}_{\text{t}}\text{)}=\text{log}\;{\text{q}}_{\text{e}}-\frac{{\text{k}}_{1}}{2.303}\text{t}$$

(7) $$\frac{\text{t}}{{\text{q}}_{\text{t}}}=\frac{1}{{\text{k}}_{2}{\text{q}}_{\text{e}}^{2}}+\frac{1}{{\text{q}}_{\text{e}}}\text{t}$$

The calculated Pseudo first-order and pseudo second-order constant parameters from raw data of Mendeley Data of Microsoft Excel in worksheet 3 were presented in Table 2.

**Table 2:** Pseudo first and second order model parameters for adsorption of basic yellow dye.

Types kaolin of adsorbents	Co (mg/L)	qe, exp. (mg/g)	Pseudo First Order Parameters			Pseudo Second Order Parameters		
			qe (mg/g)	K_1_ (min^−1^)	R^2^	qe (mg/g)	K_2_ (g/mg*min)	R^2^
Beneficiated	20	1.896	2.418	−0.048	0.720	2.632	0.010	0.926
Raw	20	1.842	2.027	0.053	0.729	3.086	0.005	0.869
Calcinated	20	1.742	1.991	0.051	0.645	4.111	0.0028	0.574

### 2.8 Thermodynamic behavior

The raw data of thermodynamic was presented in Mendeley Data of Microsoft Excel in worksheet 3. The thermodynamic property was used to know the exothermic and endothermic of batch adsorption for the experimental data in terms of Gibb's free energy, enthalpy and entropy a function of temperature. The raw data of effect of temperature on adsorption process was presented Mendeley Data of Microsoft Excel in worksheet 1. The values of standard change Gibbs free energy, enthalpy and entropy were obtained from according to the equations (8), (9) [5].

(8) $${\text{ΔG}}^{\text{0}}=\text{Δ}{\text{H}}^{\text{0}}-{\text{TΔS}}^{\text{0}}$$

(9) lnKc=−ΔG0RT=ΔS0R−ΔH0RT

Where ΔG^0^ = Standard change free Gibbs energy (kJ mol^−1^), $\text{Δ}{\text{H}}^{0}$ = Standard change enthalpy (J mol^−1^), $\text{Δ}{\text{S}}^{0}$ = Standard change entropy (J. mol^−1^K^−1^) and R = Universal gas constant (8.314 J mol^−1^K^−1^). Kc, the equilibrium constant, represents the ability of the adsorbent to retain the adsorbate and extent of movement of the adsorbate within the solution. The Kc is the ratio of the equilibrium concentration of the dye (qe) attached to adsorbent compared to the Van't Hoff equation as equilibrium dye concentration in solution (Ce). The calculated values of standard change Gibbs free energy, enthalpy and entropy constant parameters from raw data of Mendeley Data of Microsoft Excel in worksheet 3 were presented in Table 3.

**Table 3:** Thermodynamic parameters of adsorption of basic yellow dye using kaolin adsorbents.

Types of adsorbent	${\text{ΔG}}^{\text{0}}$ [KJ.Kmol^−1^]			${\text{ΔH}}^{\text{0}}$[KJ.Kmol^−1^]	${\text{ΔS}}^{\text{0}}$[KJ.Kmol^−1^K^−1^]
	Temperature [K]				
	303.15	323.15	343.15		
Beneficiated Kaolin	−1.243	1.576	4.396	−43.989	−0.141
Raw Kaolin	−0.414	1.913	4.240	−35.700	−0.116
Calcined Kaolin	0.980	3.087	5.193	−30.953	−0.105
